# What does the literature tell us about health workers' experiences of task‐shifting projects in sub‐Saharan Africa? A systematic, qualitative review

**DOI:** 10.1111/jocn.13349

**Published:** 2016-06-23

**Authors:** Hana Mijovic, Jacob McKnight, Mike English

**Affiliations:** ^1^ London School of Hygiene and Tropical MedicineLondonUK; ^2^ McMaster University Children's HospitalHamiltonOntarioCanada; ^3^ Oxford Health Systems Collaboration – Africa (OHSCAR)Nuffield Department of MedicineUniversity of OxfordOxfordUK; ^4^ KEMRI Wellcome Trust Research ProgrammeNairobiKenya; ^5^ Nuffield Department of MedicineUniversity of OxfordOxfordUK

**Keywords:** task‐shifting, sub‐Saharan Africa, neonatal nurseries, capacity issues, neonatal care, nursing workforce

## Abstract

**Aims and Objectives:**

To review systematically, qualitative literature covering the implementation of task shifting in sub‐Saharan Africa to address the growing interest in interventions of this kind. This review aims to distil the key practical findings to both guide a specific project aiming to improve the quality of neonatal care in Kenya and to contribute to the broader literature.

**Background:**

Task‐shifting programmes aim to improve access to healthcare by delegating specific tasks from higher to lower skilled health workers. Evidence suggests that task‐shifting programmes in sub‐Saharan Africa may improve patient outcomes, but they have also been criticised for providing fragmented, unsustainable services. This systematic review of qualitative literature summarises factors affecting implementation of task shifting and how such interventions in sub‐Saharan Africa may have affected health workers' feelings about their own positions and their ability to provide care.

**Design:**

Following literature search, a modified Critical Appraisal Skills Program (CASP) framework was used to assess quality. Thereafter, analysis adopted a thematic synthesis approach.

**Methods:**

A systematic literature search identified qualitative studies examining task ‐shifting interventions in sub‐Saharan Africa. Thematic synthesis was used to identify overarching themes arising from across the studies and infer how task‐shifting interventions may impact on the health workers from whom tasks are being shifted.

**Results:**

From the 230 studies screened, 13 met the inclusion criteria. Overarching themes identified showed that task shifting has been associated with jurisdictional debates linked to new cadres working beyond their scope of practice, and tension around compensation and career development for those taking on tasks that were being delegated.

**Conclusions:**

Based on the qualitative data available, it appears that task shifting may negatively impact the sense of agency and the ability to perform of health workers' from whom tasks are shifted. The potential implications of task shifting on all health workers should be considered prior to implementing task‐shifting solutions.

**What does this paper contribute to the wider global clinical community?**

- We find that task‐shifting planners should be mindful of existing professional identities and that all interventions should be designed in co‐ordination with the cadres affected by the changes made. We also find that task shifting in low‐resource settings is almost always associated with new cadres rapidly extending their roles and so adequate support, supervision and structure must guide implementation. Finally, we suggest that policy planners investigate healthcare structures including pay scales, and career development for new cadres while weighing up other potential interventions.
- This review helps inform the design of task‐shifting implementation efforts that aim to address global health workforce shortages. In particular, it draws together the available literature to derive guidance on implementing task‐shifting interventions so that they relieve pressure on nurses and other health workers.

## Introduction

### Task shifting in Nairobi's neonatal nurseries

The provision of high‐quality care to sick newborns presents a challenge to all health systems. To meet basic needs they must be kept warm and fed; staff must observe strict infection prevention practices; monitor them regularly for deterioration; and counsel and instruct families in care. Whereas for stable preterm or low birth weight babies these needs may be best met by Kangaroo Mother Care, when clinically unstable such needs, together with other technically involved clinical interventions, are typically provided by healthcare staff. This is why, in countries such as the UK, even for babies who do not require intensive care, guidelines suggest that there should be one nurse for every two to four sick babies (BAPM 2001, NANN 2009) with evidence suggesting a relationship between lower nurse ratios and higher mortality in high‐income settings (BAPM 2001).

In low‐resource settings, the need for greater provision of nursing care to newborns is stark. While enumerating the lack of neonatal nursing provision in Nairobi is an aim of the project of which this literature review is a part, initial interviews with experts in the field suggest that a single nurse may look after 20–40 babies (unpublished data) – 10 times the UK recommendation. Importantly, although the shortage of staff in this area is well known, the Kenyan government is being challenged to provide increased levels of care across the entirety of the health system. However, the provision of a much greater number of nurses to provide neonatal care is very unlikely. Whereas recent data suggest that Kenya has more than 50,000 nurses registered to practice, fewer than 17,000 offer care in the public sector that is most relied on by the poor for inpatient care (Wakaba *et al*. 2014). Thus, the main factor constraining expansion of the nursing workforce to improve access to services through the public sector in Kenya is absence of adequate finance (NCK 2014).

African governments can ill afford to miss opportunities to provide better care to newborns. Millennium Development Goal 4 (MDG4) aimed to reduce the 1990 under‐five mortality rate by two‐thirds before 2015. Kenya is one of a majority of sub‐Saharan African countries that have failed to reduce overall child mortality in line with MDG4. This disappointing result can be directly linked with the failure to reduce neonatal mortality, with absolute rates in many African and South Asian countries at least 10 higher than in developed countries. Consequently, neonatal mortality now accounts for over 40% of all child deaths in many of these countries (Lawn *et al*. 2014). Recent research suggests that although the provision of rural healthcare interventions is an important part of reducing neonatal mortality, inpatient neonatal care is also a major contributing factor and should be targeted (Moxon *et al*. 2015).

Globally, however, addressing issues relating to human resources for health and health financing have been identified as the most significant bottlenecks in the care of small and sick newborns (Moxon *et al*. 2015). At the intersection of human resource solutions that might improve both access and cost containment lies task shifting. Task‐shifting interventions should improve, rather than reduce, quality of care. In addition, we note that they are not simply technical solutions to fill service gaps, but instead a complex intervention with potentially wide effects on the health system. Given these realities, we undertook a review to provide clear and practical guidance by analysing literature covering task‐shifting projects in sub‐Saharan Africa to inform the design of possible task‐shifting solutions in neonatal care in Kenya and other low‐income countries.

### Task shifting in sub‐Saharan Africa

We will use the World Health Organization (WHO) definition of task shifting. This is, ‘the rational redistribution of tasks among health workforce teams', wherein ‘specific tasks are moved, where appropriate, from highly qualified health workers to health workers with shorter training and fewer qualifications in order to make more efficient use of the available human resources for health' (WHO 2008).

Task‐shifting interventions have a long history in sub‐Saharan Africa spanning nonphysician clinicians to community health workers, but gained prominence as a way to scale up and decentralise HIV care (WHO 2008) with growing importance in other specific service areas such as emergency obstetric surgery (Gessessew *et al*. 2011) and mental health (Bhana *et al*. 2010). The principle of delegating tasks itself is, of course, not new. Task shifting has been occurring informally in response to shortage of human resources across various settings, be it an epidemic outbreak or an ongoing coping mechanism at an understaffed health facility (Lehmann *et al*. 2009).

That task‐shifting results in no diminution of quality while improving access is supported by a number of systematic reviews and meta‐analyses. Quality of HIV care provided by adequately trained and supported nurses is comparable to the quality of care provided by physicians (Kredo *et al*. 2014). Nonphysician health workers can effectively manage noncommunicable diseases in the community, although authors of the review pointed out that further research is needed (Joshi *et al*. 2014). Specific surgical procedures such as voluntary male circumcision have been performed safely by nonsurgeons (Ford *et al*. 2012). Task shifting can also help reduce healthcare costs. A recent review of economic evaluations suggested that task shifting in low‐income countries may increase the number of services provided at a given quality and cost (Fulton *et al*. 2011).

Task shifting within a neonatal unit caring for highly vulnerable patients may, however, pose particular challenges even though the WHO suggests this solution (WHO 2012) and the Essential Care of Small Babies (ECSB) training programme incorporates task shifting of basic skills such as nasogastric feeding to mothers where plausible (Moxon *et al*. 2015). Task‐shifting programmes have, however, been criticised for being conducted in a vertical manner with insufficient attention to the complexities of health systems leading to ineffective provision of services and concerns about long‐term sustainability (Lehmann *et al*. 2009). In addition, health professionals, including those represented by the World Health Professions Alliance (WHPA) have expressed strong concerns about the manner in which task shifting is implemented and potential implications for health workers (WHPA 2008). It is therefore important to understand how task shifting affects not only the patients but also the health workers and health systems in which they operate.

Studies of task‐shifting interventions to date have focused primarily on quantitative evaluations of patient outcomes and proficiency of new cadres of health workers. Less is known about the broader effects of task shifting, including the experiences of professional and lay health workers implicated in delegation of tasks (Colvin *et al*. 2013, Yaya Bocoum *et al*. 2013). A Cochrane qualitative literature review published in 2013 examined implications of task shifting for lay health workers (Glenton *et al*. 2013), but no systematic review of qualitative studies examining existing health workers' experiences with task shifting in sub‐Saharan Africa has been published to date.

## Aims

The overall aim of this qualitative literature review is to contribute to the understanding of how task‐shifting interventions operate in the resource‐limited settings most commonly found in sub‐Saharan Africa. We want to understand the possible intended and unintended outcomes of task‐shifting interventions for the various stakeholders involved.

Specifically, this review seeks to answer the following:

- How have task‐shifting interventions in sub‐Saharan Africa influenced existing health workers' sense of agency and ability to provide care?
- Based on the literature review what recommendations can be made for task shifting as a potential intervention in Nairobi's neonatal nurseries, in addition to the general, global guidelines provided by the WHO and the WHPA?

## Methods

### Design

We planned *a priori* to present findings in a narrative format, rather than summarised as quantitative data in keeping with our overall aims. Our literature search criteria were kept relatively broad, but only studies from sub‐Saharan Africa were considered to capture experiences from resource‐limited settings similar to the future task‐shifting project environment in Kenya.

PubMed, Embase and CINAHL databases were searched for terms: ‘task shifting'; ‘task sharing'; ‘task delegation'; ‘task substitution' or ‘delegation of work' in all countries of sub‐Saharan Africa, with additional parameters set to capture qualitative and mixed‐methods studies. No time period was set. In addition, grey literature and secondary references were searched. Due to difficulties with conducting detailed content analysis in multiple languages, only English language studies were considered, which may have excluded studies from Francophone and Lusophone countries. The full literature search strategy is available in Appendix Table A1. The research was approved by the London School of Hygiene ethics committee.

Inclusion criteria:

- Qualitative or mixed‐methods studies conducted in sub‐Saharan Africa.
- Formal and informal task‐shifting interventions in healthcare involving delegation of tasks from one cadre of health workers to another cadre.
- Studies providing primary, qualitative data regarding experiences with task‐shifting interventions by national policy makers, health managers, health workers and/or healthcare recipients.

Exclusion criteria:

- Nonprimary data (i.e. policy briefs, opinions, progress reports, systematic reviews).
- Mixed‐methods studies where the qualitative component was deemed insufficient to contribute to further analysis.
- Studies focusing on task shifting of a very specific intervention rather than a broader set of tasks (i.e. initiation of antiretroviral therapy only, rather than management of HIV patients).

### Search outcome

**Figure 1:**
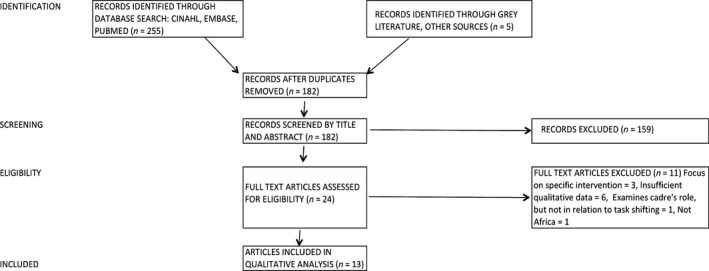
PRISMA flow chart.

From 230 studies identified, 13 studies met the inclusion criteria and were included in the review. The literature search process is summarised in the PRISMA Flow chart in Fig. 1.

### Quality appraisal

Numerous appraisal tools have been developed for qualitative studies (Thomas & Harden 2008, Hannes 2011). We used the Critical Appraisal Skills Program (CASP) quality assessment tool (CASP UK 2013) with some modifications made to reflect the character of studies under review. Modified CASP criteria have been used frequently in qualitative reviews of health interventions, including a review of task delegation to lay health workers by the Cochrane Collaboration (Glenton *et al*. 2013), which we referred to in order to modify our checklist.

Quality appraisal based on a modified CASP checklist is provided in Table 1. The amount of qualitative data available was limited by the fact that most studies were relatively brief and relied primarily on one‐time interviews and focus group discussions (FGDs). Most of the studies did not employ additional qualitative methods such as serial in‐depth interviews, field observations or examining documents produced by health workers. Nevertheless, a number of studies provided rather diverse data, accounting not only for commonly expressed views but also for contradictory perspectives and opinions conveyed by the informants. Although all the studies detailed their recruitment strategy, none of the studies fully accounted for informants who refused to participate or explored how informant selection influenced study findings. Many studies explicitly stated that the study research was conducted as part of a task‐shifting programme evaluation, but most researchers did not reflect on how informants' responses or subsequent data analysis may have been influenced by the role of the research team.

**Table 1:** Quality appraisal checklist (CASP)

	Appraisal criteria	Yes	Somewhat	No
1	Is this study based on qualitative, narrative research?^a^ For mixed‐methods studies, is there sufficient emphasis on the qualitative component?	13	0	0
2	Are the study context and objectives clearly described? Study setting adequately described? Rationale for conducting the study stated and justified?	13	0	0
3	Is there evidence of researcher reflexivity? Researcher's role, potential bias and influence on respondents examined in formulation of questions, data collection and data analysis?	1	3	9
4	Is the recruitment strategy appropriate to the study aims? Researcher explained how study informants were selected? Discussion around recruitment, i.e. why some people chose not to take part?	1	12	0
5	Is the method of data collection clearly described and appropriate for the research question? Data collection method explicitly stated? Saturation of data discussed?	5	8	0
6	Is the data analysis sufficiently rigorous? Analytic process described in sufficient detail? If thematic analysis is used, is it clear how themes/categories were derived? Are contradictory data taken into account?	11	1	1
7	Are conclusions supported by sufficient evidence? Did the data provide sufficient depth, detail and richness? The researcher discussed credibility of their findings (triangulation, respondent validation, more than one analyst)?	8	4	1

a Screening question, captured in inclusion criteria.

These observations are not unique to our literature review. As pointed out by Glenton *et al*. (2013) in a Cochrane qualitative literature review, qualitative articles published in journals tend to provide relatively ‘thin' data and are less likely to include a variety of data gathering methods. Glenton and others also reported lack of researcher reflexivity as a common finding when qualitative studies are being appraised. Longitudinal, ethnographic research may be better suited to qualitative studies that examine health interventions (Pawson *et al*. 2005, Glenton *et al*. 2013, Dawson *et al*. 2014), but such research is more time and resource demanding and often too extensive to be published in widely circulated health research journals.

Thus, all the studies meeting the original inclusion criteria were included in the subsequent analysis regardless of the quality score assigned. Although some studies were deemed to be of lower methodological quality, the insights from stakeholders they presented nevertheless contributed to the richness of data and were informative for data synthesis. This is one of the approaches commonly adopted in qualitative reviews, especially when there are a limited number of studies available (Pawson *et al*. 2005, Hannes 2011).

### Data abstraction

Following Thomas and Harden (2008), a thematic synthesis approach was used to compile the data. In following this approach, it is important to note the objective of this review – to inform the study of task shifting for work being developed in Kenya. Given this aim, we were interested in ‘key concepts' (Campbell *et al*. 2003) that might illuminate the characteristics of effective task‐shifting programmes while highlighting the major barriers to implementation. Of course, we also needed to remain true to the texts we examined, all of the noted facets of implementation and the character of the reformed systems studied. In this way, although our aims were pragmatic and directed towards the needs of our future project, we were also aiming to provide as much ‘thick description' as possible (Geertz 1973) and so the search was not governed by the need for direct or concise ‘answers'.

Text was manually coded, and organised under initial descriptive themes. These themes were iteratively improved through discussion between the reviewers. Due to the paucity of qualitative research on task shifting in sub‐Saharan Africa, there was a great deal of variety between texts, and so line‐by‐line coding would have been tedious and potentially distracting. As such, codes were generated inductively and organised under 29 ‘descriptive themes' (Thomas & Harden 2008). A table showing the listing of these original descriptive themes is included in Appendix Table A3.

### Synthesis

To move beyond simple description and towards theory, the descriptive themes were then subjected to a further round of analysis. Again, following Thomas and Harden (2008), the aim was to generate ‘analytical themes'. Here, it was also possible to reintroduce the aims of the overall project – to derive findings that will guide future research. This approach follows the Cochrane Collaboration advice on synthesis that advises that the type of analysis pursued should reflect the research question (Noyes & Lewin 2011). Accordingly, the themes were addressed from the perspective of a programme implementer: how could the thematic content be best summarised so as to be most useful and descriptive to someone considering task shifting? This simple approach helped to reorganise the findings in a very pragmatic way. Perhaps naturally, the end result was not a list of ‘themes', but rather a list of ‘synthesis statements' that, we feel, speak directly to policy makers. This process resulted in three core synthesis statements, with eight underlying explanatory synthesis statements.

## Results

### Characteristics of included studies

A detailed description of included studies is provided in Table 2, with full reference list attached in Appendix Table A2. In summary, 12 of 13 studies were relatively brief articles published in health and social sciences journals and one study was a PhD thesis. All except for one study were published within the last three years, which speaks to the fact that investigation of formal task shifting is a relatively new phenomenon. Studies covered a broad range of task‐shifting interventions delivered through secondary and primary health facilities as well as community outreach work. The majority of the respondents were policy makers, facility managers and health workers, with fewer studies including perspectives of healthcare recipients. Although most of the studies examined formal task‐shifting interventions, some explored informal task shifting. Most of the data were obtained through in‐depth, semi‐structured interviews and focus group discussions (FGDs).

**Table 2:** Study summaries table

#	First author, year (country)	Sector	Facility level	Study type	Cadres involved	Tasks shifted	Formal or informal	Informants	Data collection methods	Key findings by authors
1	Baine (2014) (Uganda)	Multi‐sector	All levels	Health workforce analysis	All	Variable	Informal	Policy makers, CSOs	Interviews	Lower cadres perceived as incompetent and overworked Need for formal policy/support Task shifting perceived as expensive relative to supporting existing workforce
2	Callaghan‐Koru (2012) (Malawi)	Primary care, Paediatrics	Community outreach	Programme evaluation	Clinic nurses and midwives to CHWs	Curative, preventative, data collection	Formal	Facility managers, CHWs	FGDs, Interviews	Expanded access Reducing caseloads at health facilities Contrasting views on scope of CHW work Frustration on system constraints and community expectations
3	Cataldo *et al*. (2015) (Zambia)	HIV	Primary, Community outreach	Health workforce analysis	Clinic nurses to community care givers	Treatment, monitoring	Formal	CBOs, CHWs	Interviews, observation	Shift from support to specialised tasks Lack of formal recognition, remuneration, mobility Shifting relationship with patients and communities
4	Cumbi (2007) (Mozambique)	Surgery, Obstetrics	Secondary, Primary	Programme evaluation	Physicians and nurses to Surgical Assistants	General surgery, emergency obstetrics	Formal	Facility managers, physicians, nurses	FGDs, interviews	Health workers satisfied with surgical assistants' work Life‐saving skills Reduced need for referral Lower cost to patients and families Inadequate remuneration
5	Dambisya (2012) (Uganda)	Multi‐sector	All levels	Health workforce analysis	All	Variable	Informal	Policy makers, Facility managers, Health workers, nursing students	FGDs, interviews	Front‐line workers held misconceptions with regards to meaning and intention of task‐shifting Task shifting widely accepted in HIV care No policy to guide task shifting, but environment conducive Task shifting can address lack of skills and need for services Task shifting already happening, needs regulation Task shifting as quick fix for the poor Task shifting compromising quality of care and may compromise the health system
6	Ferrinho (2012) (Mozamnique and Zambia)	Multi‐sector	Secondary, Primary	Health workforce analysis	All	Variable	Informal	Facility managers, health workers, auxiliary staff	FGDs, interviews	Healthcare workers have to practice beyond their scope of practice to cope with daily tasks Healthcare workers perform tasks they deem necessary without formal instructions Assuming additional tasks is not reflected in job descriptions, remuneration or career progression Ancillary staff and nurses assume the greatest variety of new functions
7	Ledikwe (2013) (Botswana)	HIV	Primary, HIV clinics	Programme evaluation	Nurses to lay counsellors	Counselling, administrative	Formal	Policy makers, Facility managers, health workers, lay counsellors, patients	Interviews, observation	Lay counsellors generally comfortable with work tasks, but some expected to work beyond scope of training/mandate Lack of resources, support, supervision
8	Mpofu (2014) (Botswana)	M&E	Secondary, Primary	Programme evaluation	Nurses to M&E officers	Data collection and reporting	Formal	Policy makers, Health facility managers, M&E officers	FGDs and interviews	Improved M& E capacity Increase utilisation of health data More time for nurses and other health workers to focus on clinical duties Importance of clarifying role of new cadres Need to align resources with expectations Sustainability
9	Munga (2012) (Tanzania)	Multi‐sector	Secondary, Primary	Health workforce analysis	All cadres	Variable	Formal and informal	Policy makers, national‐level managers, CSOs	Interviews	Task‐shifting long‐standing coping mechanism Task shifting compromised quality of care if not supported by appropriate infrastructure Task shifting increases access to services in remote areas Task shifting may lead to professional discontent Need for additional training and supervision
10	Ochieng (2014) (Kenya)	Primary care	Community outreach	Health workforce analysis	Clinic nurses and midwives to CHWs	Curative, preventative, data collection	Formal	Policy makers, heath facility managers, health workers, patients	FGDs and interviews	Commonly shifted tasks promotive, preventative, simple curative. Common motivations: supportive supervision, training, identification, resources, training, recognition, community dialogue Some health workers assumed curative tasks beyond task‐shifting mandate due to patient demand, economic hardship Curative tasks demand further training and regulations
11	Smith (2014) (Malawi)	Primary care	Community outreach	Health workforce analysis	Clinical nurses to CHWs	Curative, preventative, data collection	Formal	Policy makers, Community supervisors, CHWs	FGDs, interviews, observation, documents	CHWs performed variety of tasks in addition to those in job description Overloading, specialisation, competing demands, role confusion, shifting from initial role Lack of adequate training, resources, supervision
12	Spies (2014) (Ethiopia, Kenya, Tanzania, Uganda)	Multi‐sector	All levels	PhD Thesis	Doctors to nurses	Variable	Formal and Informal	Nurse leaders	Interviews	Nurse burden in the presence of health worker shortages and WHO push for task shifting Need for site and task‐specific education Need for policy and regulatory support Need for clearly defined scope of practice
13	Yaya Bocoum (2013) (Burkina Faso)	HIV	Secondary, Primary	Health workforce analysis	Doctors to nurses Nurses and midwives to auxiliary midwives,CHWs	Clinical care and counselling.	Formal	Policy makers, Members of professional associations, health workers	Interviews	Task shifting has impact on health system as a whole Some short‐comings inherent to task shifting and others reflective of broader health system issues Increased sense of responsibility and worthiness among health workers Increased satisfaction with newly acquired skills Improved patient–provider relationships Staff frustration with lack of resources

### Synthesis statement 1

Successful task‐shifting interventions are mindful of the professional jurisdictions of the staff who will be affected by the planned change and design the intervention in co‐operation with them.

#### Category 1 – The professions involved must be aware of the need for a change, and their own role and professional identity should not be diminished as a result of the reform

Task‐shifting programmes introduced new professional and lay cadres of health workers, or changed the job roles of existing cadres. It should perhaps be obvious that such changes resulted in *jurisdictional* tensions between the professionals affected (Abbott 1988). An overarching theme emerging from both senior and frontline staff was the sentiment that the role of doctors and nurses in the healthcare system was being diminished through the task‐shifting process. The mechanisms attributed to the role erosion included pushing highly skilled professionals out of the workplace (Study #1, #4, #5, #9), changes to one's workload and work role (Study #3, #11, #12) and allowing for suboptimal quality of healthcare (Study #1). Although the specific categories of workload and suboptimal care are described in the next sections, it is important to remember that, more generally, the professions affected by the reform must be an active component of the change process rather than being alienated from it.

#### Category 2 – The intervention must result in a manageable workload for all affected staff

Task shifting was widely welcomed and acceptable when it involved delegation of nonclinical tasks, including data collection, administrative work, ensuring treatment compliance and patient counselling. Health professionals felt that this kind of task shifting enabled them to focus on their ‘real' work including clinical tasks and managerial duties. Introduction of a Monitoring & Evaluation (M&E) cadre in Botswana provided a particularly good example of a task‐shifting intervention that health workers perceived as overwhelmingly beneficial to their work:

So, when the district M&E officers came in, they relieved the community health nurse in such a way that the community health nurse is able to go to facilities to attend to such programmes as child health and others. The district M&E officer then took up [data responsibilities] for different HIV programmes. (District Manager, Botswana, Study # 8)

When it came to delegation of clinical tasks, experiences of doctors and nurses were mixed. Nurses interviewed in the studies frequently found themselves in a ‘bottleneck' position where new tasks were being delegated to them, whereas they had no one to whom they could offload some of their duties. Although many nurses appreciated the opportunity to learn new skills when tasks are shifted to them, this often came at a price of increased workload, inadequate supervision and inability to perform what they perceived to be their ‘core' nursing duties. Some nurses felt that taking on new tasks effectively meant they were ‘shifting away' from the nursing profession:

We shift from the nurses' profession … we can't make a person, a single person to do many tasks. (Nurse Leader, Ethiopia, Study # 12)

Whereas many nurses felt that task shifting had a negative impact on their work load and work role, lower skilled cadres who assumed nurses' work generally felt that task shifting benefited nurses and strengthened workplace relationships:

Our working relationship with nurses in government health institutions has grown and is enhanced because we do almost similar work […] we make work lighter for health workers. (Home‐based caregiver, Zambia, Study # 3)

Data about changes to workload and work role of doctors were rather limited. Some doctors felt that delegating their tasks to nurses or technical assistants lessened the workload and contributed to a sense of trust and team building. Others felt that task shifting threatened their scope of work and their authority because lower cadres were given too much autonomy or were unwilling to collaborate:

… Yes! The workload has decreased, but also it makes you feel trusted! Because when you work with someone and you delegate certain tasks, the person feels appreciated and they do their work well! Consequently our relationship keeps improving. (Medical Doctor, Burkina Faso, Study #13)

…There is no space… the medical doctors who went to safe motherhood training programme for obstetric care do not make use of this training, due to lack of collaboration … (Medical Doctor, Mozambique, Study #4)

#### Category 3 – The intervention must work on a ‘First do no Harm' basis, ensuring that the cadre's work is clinically effective, and is accepted as ethical by the supporting cadres

Along with potentially diverting the resources away from higher skilled professionals, task shifting was also perceived to be eroding the quality of the healthcare provided. Although task shifting was at times recognised as an inevitable measure to meet healthcare demands at hand, it was also seen as a threat to the standard of care that doctors, nurses and midwives had aspired to provide, albeit under resource‐limited circumstances. Task shifting was therefore frequently met with some degree of cynicism and apprehension:

When we hear the word task shifting in Kenya … our hair stands out straight. The word has been used around the world, especially in the developing world to promote that you are going to use very low qualified cadres … (Nurse Leader, Kenya, Study # 12)

This last quote from Spies (2014) is of particular note for task shifting within clinical environments as it highlights the need for a style of introduction that embraces nurse opinions and uses them to design more effective projects.

### Synthesis Statement 2

TS in low‐resource settings is almost always associated with overstretch of roles and consequent unethical practice and so adequate support, supervision and structure must guide an appropriately scaled intervention.

#### Category 1 – Task‐shifting programmes should be accompanied by substantial training efforts and supported by strong supervision and complementary practice from related cadres

Studies researching formal task‐shifting programmes almost unanimously highlighted the discrepancy between the roles of ‘new' health workers as envisioned by the programme planners and the actual roles performed. New cadres in particular had the tendency to assume additional tasks that were not originally envisioned for their role. What was introduced as a formal task‐shifting process therefore commonly extended into informal task shifting under field conditions. This was facilitated by limited professional regulations as well as inadequate or nonexistent job descriptions and supervisory mechanisms noted by many studies.

I might be a lay counsellor, but I have really been taught nursing duties by the nurses I work with. I take vital signs, I dispense medication, I dress wounds and even write reports for the nurses. Sometimes I spend more time doing their work than mine. (Lay Counsellor, Botswana, Study # 7)

Not all the works we do were written on the job description because there are other organisations which use us. So we cannot say that we only follow what was written to us by the government. (CHW, Malawi, Study # 11)

Narratives by health workers across the studies showed that health workers commonly perform tasks beyond their scope of practice to cope with workplace demands and to fulfil the obligation they feel towards their patients.

Clearly, this fundamental risk should be considered as part of any task‐shifting intervention. Project planners need to recognise that any task‐shifting programme is limited by the health system of which it is a part. Accordingly, the intervention must be designed to provide supervision of staff to ensure that they are not stretching the mandates of their new or altered job roles. Staff who are working in positions affected by the intervention should also be trained to be mindful of the limitations of the redesigned structure. Specifically, for interventions in such areas as neonatal care, nurses and paediatricians should be trained to understand the limits of the new cadre, and to ensure that they remain supportive of the new staff, but also watchful of their activity.

#### Category 2 – Task‐shifting programme design should be mindful of the perspective of patients and ensure that key differences in cadre are understood

Many health workers conveyed that their patients could not fully tell the difference between doctors, nurses or lay workers. A commonly held perception was that patients either were not aware or did not mind that tasks were being delegated to lower cadres.

In the past people (in the villages) used to call us doctors, but with this programme we are real doctors because we are giving them medicines and I feel happy that I am a doctor. (CHW, Malawi, Study # 2)

The inability of patients to recognise the difference between health workers is an insight that should not be disregarded. While this fact may mean that patients in some areas appear willing to receive care from new cadres, it also means that patients may not be able to recognise when care is delivered inappropriately – a reality of the majority of the task‐shifting programmes studied. Other studies suggested that patients were naively accepting care from lower skilled workers while believing that they were being looked after by a professional. Views conveyed by representatives of ‘health service consumers' in Uganda were summarised as follows:

Patients were reportedly uncomfortable and dissatisfied with being handled by low cadre health workers. They wanted to be handled by doctors. They felt bad on learning that the attending health worker was not a doctor. (Uganda, Study #1)

Ideally, the views of health workers and policy makers would be triangulated with the views of patients themselves. In the studies reviewed, views of patients and communities were largely conveyed through the interviews with health staff, managers and policy makers, and therefore provided a rather limited insight. The lack of patient voice captured in the studies reviewed is a significant weakness in the literature and any intervention in neonatal care should be mindful of the role and opinions of mothers of patients (e.g. Coulter *et al*. 2014).

### Synthesis Statement 3

The structure of the health system into which the TS project is introduced should be considered for relative pay scales, career development and potentially better alternatives to task shifting.

#### Category 1 – To avoid tensions between cadres and illicit charging for services, pay levels must be equitable and adequate

Health workers involved in task shifting ranged from local volunteers who received little to no monetary compensation to nurses whose salaries were regulated at the national level. In many studies cadres participating in task shifting assumed higher workload and increased level of responsibility than anticipated, but this was usually not reflected in their remuneration. Managing the expectations of workers involved in task shifting, or affected by it, is essential because where staff feel they are not adequately paid, undesirable outcomes are noted.

We expected that after being trained, since we are now part of the curative part, there will be change in our monthly salaries but there is no change … (CHW, Malawi, Study # 2)

Although tasks performed by lower skilled health workers were not always paid through formal mechanisms, some health workers found ways to ‘cash in' on their tasks. A study among CHWs in Kenya alluded to volunteer, periurban workers who wished to ‘*acquire skills to sell, because of hard economic times in the country*' (Study # 10). Surgical Assistants in Mozambique were known, at times, to be ‘obliged to ask for illicit charges' because of low remuneration relative to the amount of work they performed (Cumbi *et al*. 2007). Anecdotally, training lay workers in the community in some cases also resulted in untrained individuals posing as health workers and charging for their services (Study # 2, 10, 11).

#### Category 2 – TS should not be initiated in a health system where existing solutions are available and affordable

In certain settings task shifting was seen as a direct threat to job safety and future employment prospects for nurses and doctors. In Uganda (Study # 1, 5), where a considerable number of doctors and nurses were unemployed or working overseas, task shifting was perceived by some health professionals and policy makers primarily as a short‐sighted, cost‐saving strategy, effectively pushing established professionals out of the healthcare system.

That [task shifting] is an anomaly Uganda cannot afford. As long as we need the professionals, and they are within the country, we should employ them … (Senior Manager, Uganda, Study # 5)

Studies in the review highlighted the tension between health workers assuming new tasks who expected adequate compensation for the work performed and the policy makers who commonly assumed that task shifting was a ‘cost saving' strategy. This is an important insight for any task‐shifting programme in Kenya that affects nurses. There are many thousands of unemployed nurses in Kenya, and the benefits and limitations of the introduction of a new cadre in neonatal care should be compared to the prospect of employing more nurses in this area.

#### Category 3 – TS interventions must allow for career planning of all affected cadres

Higher skilled cadres, managers and policy makers frequently agreed that compensation of new, lower cadres and opportunities for career progression were inadequate and could potentially compromise the long‐term success of task‐shifting programmes. The quotes below refer to the newly introduced cadres of M&E Officers and Surgical Assistants respectively:

They are watching their colleagues moving up the ladder! They are just in one place. Even the good ones … we are going to lose them if this trend continues. (Program Officer, Botswana, Study # 8)

… An individual spends six years in school and continues to be considered mid‐level [it's unjust]… There is a huge gap between the salaries of medical doctors and the TCs…[surgical assistants]. (Medical Doctor, Mozambique, Study # 4)

At the same time lower skilled cadres were often seen as part of the solution to providing healthcare to underserviced areas. They had good retention rates compared to higher skilled staff and they came at a substantially lower cost. It was widely acknowledged that lower, less skilled cadres performing tasks at a lower cost was in fact what made task shifting a plausible mechanism for providing additional health services in the first place:

Skills of lower cadre health workers and especially community health workers are hardly portable both nationally and internationally. Lower cadre health workers can also be easily and cheaply recruited from within areas where they live and where they are supposed to be working. It is thus easy to retain these workers as they are already used to the living conditions of their localities. (District Level Informant, Tanzania, Study # 9)

Contrasting the findings associated with lower and higher levels of task shifting, it appears that structured career planning is more of an issue for skilled staff taking on new tasks. With that said, lower‐level staff involved in task shifting, especially new lower cadres such as that envisioned in the Kenyan scheme, seem likely to view their training as an opportunity to become recognised providers of medical care. To prevent lower cadres being tempted to enact informal charging or to misrepresent themselves as nurses or doctors, lower cadres should be closely monitored and adequately paid. In addition, although this is less of a concern for lower‐level workers, their formal position within the hierarchy of healthcare positions should be planned, and the requirements for entry to more advanced posts made clear.

## Discussion

### Limitations and strengths

#### Defining task shifting in literature search

Task‐shifting interventions may not be labelled as such in literature. For example, systematic review of midwifery services found that although the term ‘task shifting' was used commonly in relation to community health workers, ‘task shifting' was used infrequently when describing interventions involving midwives (Colvin *et al*. 2013). Our literature search included terms that were synonymous/near synonymous with task shifting as well as a review of secondary references. The list of search terms was not exhaustive and it is possible that the studies identified were more likely to represent some cadres than others.

#### Obtaining rich qualitative data

As mentioned in the discussion on the quality of studies included in the review, qualitative studies published in health journals provide a diverse, but somewhat limited amount of data.

Further grey literature searches with focus on obtaining unpublished documents from various health organisations and identifying extensive ethnographic projects conducted by anthropologists would potentially provide richer data and inform subsequent analysis.

#### Quality of the studies in the review

Studies were included regardless of the quality score assigned. All studies provided narratives that were helpful in drawing a larger picture about the impact of task‐shifting programmes on health workers. Due to limited researcher reflexivity and scant information about study informants, reliability of individual study findings was at times difficult to ascertain. It is likely that important perspectives were missed or remained unexplored by study authors. In our discussion of findings we pointed to areas for further inquiry that may allow for discerning additional opinions and experiences.

#### Reviewer bias

The first‐named author conducted the literature search, but the criteria for selection were chosen by both the first and second authors. In addition, while the analysis of the literature and subsequent synthesis statements were produced by the first two authors, the third author provided a thorough check of methods and conclusions. In this way, the potential for reviewer bias was reduced. The first and third named authors are paediatricians with interests in neonatal care. Such experience may have resulted in a bias towards clinician perspectives. Both the second and third named authors are members of the HSD‐N project, and they may have been influenced by this association.

## Conclusion

Task‐shifting interventions in sub‐Saharan Africa have expanded far beyond the HIV sector for which they were initially developed. Although most of the evidence around task‐shifting interventions is quantitative, a growing number of qualitative studies is emerging from sub‐Saharan Africa. Qualitative studies suggest that task‐shifting interventions may carry important short‐term and long‐term implications for all cadres of health workers. Findings in this review are based on a small number of relatively short studies with several methodological limitations. Based on the data available, it appears that task shifting may negatively impact health workers' sense of agency and ability to perform their work if not carefully designed. Established health professionals have been concerned that task shifting is diminishing their role in the health system. Lower cadres assuming new tasks appear to be highly motivated to meet workplace demands and provide patient care. However, assuming new tasks may be occurring at the expense of high work burden, performing tasks beyond one's scope of practice and potentially compromising patient safety. Task‐shifting programmes have often been perceived as cost‐saving interventions, but this may have contributed to unacceptably low remuneration and career progression options for health workers, especially in rural areas. On a positive note, some task‐shifting interventions appeared to have resulted in improved communication within health teams and enabled a more rational distribution of work, especially when administrative tasks were being delegated. Some of the areas warranting further qualitative inquiry include opportunity cost of task shifting for health workers, patients' experiences with task‐shifting interventions and patient's safety. Many limitations of task‐shifting programmes arise from limitations inherent to weak, poorly resourced health systems in sub‐Saharan Africa.

We acknowledge that the certainty and transferability of our literature review findings are limited by the number of qualitative studies available and methodological shortcomings of individual studies. Nevertheless, literature review allowed us to examine first‐hand experiences and challenges arising from the field and thus complemented more general recommendations and guidelines set by the WHO and other organisations.

As mentioned in the abstract, in addition to informing a broad audience of policy makers, this review aims to provide practical guidance to an ongoing project in Kenya. Task shifting as a potential intervention in Nairobi's neonatal nurseries should be evaluated based on experiences from other programmes to avoid some of the common pitfalls that occur when programme ideas are translated into practice. The merit of task‐shifting interventions should be adjudged relative to the potential impact for patients and health workers in neonatal nurseries, as well as the Kenyan health system overall. Research arising from the HSD‐N project can fill in some of the knowledge gaps in qualitative research around task shifting and patients' experiences with healthcare in sub‐Saharan Africa.

## Relevance to clinical practice

Task shifting is typically thought of by managers as a tool to lower costs of, or expand access to care. Ideally, this happens with no diminution in quality. It is becoming an attractive policy option globally particularly perhaps in low‐income settings where attention has been paid to effectiveness of task shifting through community or lay health workers – areas that have been the subject of both quantitative and qualitative systematic literature reviews. However, there have been limited efforts to explore how task shifting impacts on services and the roles of existing health professionals from the perspective of these health professionals. This systematic review highlights potentially valid concerns of health workers about ‘mission creep' of new cadres, and about how their own roles may change, as well as their recognition of potential benefits. The findings have importance for the design of task‐shifting approaches that are acceptable to and truly complement the work of existing health professionals.

## Contributions

HM, JM and ME contributed to study design and manuscript preparation. HM and JM conducted data collection and analysis.

## Funding

This work was supported by the funds from The Wellcome Trust (#097170) awarded to ME for a Senior Fellowship together with an Oxford University / Wellcome Trust Institutional Strategic Support Fund award and a Joint Funded Health Systems Research Initiatives grant (MR/M015386/1). The funders had no role in drafting or submitting this manuscript.

## Literature search strategy

**Table A1:** Quality appraisal checklist (CASP)

DATABASES SEARCHED:	Embase, CINAHL, Embase, PubMed
LAST SEARCH DATE:	07‐Aug‐15
TIME RESTRICTIONS:	None
LANGUAGES:	English only.
TERMS (in all fields):	(“task delegation” OR “task sharing” OR “task shifting” OR “task substitution” OR “delegation of work)
AND	((“semi‐structured” OR semistructured OR unstructured OR informal OR “in‐depth” OR indepth OR “face‐to‐face” OR structure OR guide) adj3 (interview* OR discussion* OR questionnaire*) OR (focus group* OR qualitative OR ethnograph* OR fieldwork OR “field work” OR “key informant”) OR (interviews as topic/OR focus groups/OR narration/OR qualitative research*))
AND	(Angola or Benin or Botswana or Burkina Faso or Burundi or Cameroon or Cape Verde or Central African Republic or Chad or Comoros or Congo or Côte d'Ivoire or Djibouti or Equatorial Guinea or Eritrea or Ethiopia or Gabon or Gambia or Ghana or Guinea or Guinea‐Bissau or Kenya or Lesotho or Liberia or Madagascar or Malawi or Mali or Mauritania or Mauritius or Mozambique or Namibia or Niger or Nigeriaor Réunion or Rwanda or “Sao Tome and Principe” or Senegal or Seychelles or Sierra Leone or Somalia or South Africa or Sudan or Swaziland or Tanzania or Togo or Uganda or Western Sahara or Zambia or Zimbabwe)

**Table A2:** Reference list of studies included in the review

1	Baine and Kasangaki (2014)
2	Callaghan‐Koru *et al*. (2012)
3	Cataldo *et al*. (2015)
4	Cumbi *et al*. (2007)
5	Dambisya and Matinhure (2012)
6	Ferrinho *et al*. (2012)
7	Ledikwe *et al*. (2013)
8	Mpofu *et al*. (2014)
9	Munga *et al*. (2012)
10	Ochieng *et al*. (2014)
11	Smith *et al*. (2014)
12	Spies (2014)
13	Yaya Bocoum *et al*. (2013)

**Table A3:** Descriptive themes

1	Baine	Professional health workers inadequately supported/threatened/pushed out by task shifting Cadres assuming new tasks work outside of their scope of work Task shifting as a coping rather than acceptable strategy Lack of appropriate supervisory mechanisms Lack of career progression options for cadres assuming new tasks Patient's safety concerns with existing task‐shifting practices Task shifting to lower cadres is inadequately communicated to patients
2	Callaghan‐Koru	Task shifting increases access to health services in underserviced areas Cadres assuming new tasks work outside of their scope of work Divergent perspectives on appropriate tasks to be shifted among stakeholders Lack of formal role recognition for new cadres Lack of adequate remuneration Lack of adequate supervisory mechanisms Lack of resources High work burden
3	Cataldo	Task shifting changes the nature of cadre's role Opportunity to acquire new knowledge Lack of remuneration Lack of career progression Increased recognition of lower cadres within formal health system High expectations from patients go unmet
4	Cumbi	Professional health workers feel inadequately supported/threatened/pushed out by task shifting Task shifting increases access to health services in underserviced areas Cadres assuming new tasks work outside of their scope of work Lack of adequate remuneration Lack of career progression Task‐shifting fosters good working relationship
5	Dambisya	Professional health workers feel inadequately supported/threatened/pushed out by task shifting Cadres assuming new tasks work outside of their scope of work Task shifting as a coping rather than acceptable strategy Task shifting increases access to health services in underserviced areas Task shifting reduces case load in clinics
6	Ferrinho	Task shifting as a coping rather than acceptable strategy Task shifting not formalised Lack of training Lack of remuneration Lack of career progression Lack of resources Health workers frustration Health workers feeling strong responsibility towards patients/community
7	Lediwke	Sense of pride and commitment to patients Task shifting changes the nature of cadre's role Lack of resources Lack of remuneration Lack of career progression Lack of supervision Lack of consistent supervision
8	Mpofu	Task shifting of nonclinical tasks allows health workers to focus on clinical work Cadres assuming new tasks work outside of their scope of work Lack of career progression Lack of remuneration Lack of resources
9	Munga	Professional health workers feel inadequately supported/threatened/pushed out by task shifting Task shifting as a coping rather than acceptable strategy Cadres assuming new tasks work outside of their scope of work Lack of resources Lack of supervision Lack of career progression Task shifting increases access to health services in underserviced areas Task shifting costly and time consuming
10	Ochieng	Divergent perspectives on appropriate tasks to be shifted among stakeholders Lack of career progression Lack of remuneration Lack of supervision Importance of supervision Potential to build trust with communities
11	Smith	Divergent perspectives on appropriate tasks to be shifted among stakeholders Cadres assuming new tasks work outside of their scope of work Task shifting changes the nature of cadre's role Lack of training Lack of supervision Lack of resources Health workers feeling strong responsibility towards patients/community
12	Spies	Task shifting changes the nature of cadre's role Task shifting not formalised Lack of resources Lack of training Patients benefit from task shifting Patients not always aware of cadre's professional credentials High work burden
13	Yaya Boucoum	Task shifting increases sense of responsibility, self confidence, utility Task shifting fosters good working relationship Lack of training Lack of resources Patients not always aware of cadre's professional credentials High work burden for lower cadres Lower work burden for higher cadres

## References

[jocn13349-bib-0001] Abbott AD (1988) The System of Professions: An Essay on the Division of Expert Labor. University of Chicago Press, Chicago, IL; London.

[jocn13349-bib-0002] BAPM (2001) Standards for Hospitals Providing Neonatal Intensive and High Dependency Care, 2nd edn, BAPM, London, UK.

[jocn13349-bib-0003] Bhana A , Petersen I , Baillie KL & Flisher AJ (2010) Implementing the World Health Report 2001 recommendations for integrating mental health into primary health care: a situation analysis of three African countries: Ghana, South Africa and Uganda. International Review of Psychiatry 22, 599–610. 21226648 10.3109/09540261.2010.536152

[jocn13349-bib-0004] Campbell R , Pound P , Pope C , Britten N , Pill R , Morgan M & Donovan J (2003) Evaluating meta‐ethnography: a synthesis of qualitative research on lay experiences of diabetes and diabetes care. Social Science & Medicine 56, 671–684. 12560003 10.1016/s0277-9536(02)00064-3

[jocn13349-bib-0005] CASP UK (2013) Critical Appraisal Skills Programme (CASP) Qualitative Research Checklist. CASP UK, Oxford.

[jocn13349-bib-0006] Colvin CJ , de Heer J , Winterton L , Mellenkamp M , Glenton C , Noyes J , Lewin S & Rashidian A (2013) A systematic review of qualitative evidence on barriers and facilitators to the implementation of task‐shifting in midwifery services. Midwifery 29, 1211–1221. 23769757 10.1016/j.midw.2013.05.001

[jocn13349-bib-0007] Coulter A , Locock L , Ziebland S & Calabrese J (2014) Collecting data on patient experience is not enough: they must be used to improve care. British Medical Journal 348, g2225. 24671966 10.1136/bmj.g2225

[jocn13349-bib-0008] Cumbi A , Pereira C , Malalane R , Vaz F , McCord C , Bacci A & Bergstrom S (2007) Major surgery delegation to mid‐level health practitioners in Mozambique: health professionals' perceptions. Human Resources for Health 5, 27. 18062808 10.1186/1478-4491-5-27 PMC2235883

[jocn13349-bib-0009] Dawson AJ , Buchan J , Duffield C , Homer CS & Wijewardena K (2014) Task shifting and sharing in maternal and reproductive health in low‐income countries: a narrative synthesis of current evidence. Health Policy Plan 29, 396–408. 23656700 10.1093/heapol/czt026

[jocn13349-bib-0010] Ford N , Chu K & Mills EJ (2012) Safety of task‐shifting for male medical circumcision: a systematic review and meta‐analysis. Acquired Immune Deficiency Syndrome 26, 559–566. 22112602 10.1097/QAD.0b013e32834f3264

[jocn13349-bib-0011] Fulton BD , Scheffler RM , Sparkes SP , Auh EY , Vujicic M & Soucat A (2011) Health workforce skill mix and task shifting in low income countries: a review of recent evidence. Human Resources for Health 9, 1. 21223546 10.1186/1478-4491-9-1 PMC3027093

[jocn13349-bib-0012] Geertz C (1973) The Interpretation of Cultures: Selected Essays. Basic Books, New York, NY.

[jocn13349-bib-0013] Gessessew A , Barnabas GA , Prata N & Weidert K (2011) Task shifting and sharing in Tigray, Ethiopia, to achieve comprehensive emergency obstetric care. International Journal of Gynaecology and Obstetrics 113, 28–31. 21315350 10.1016/j.ijgo.2010.10.023

[jocn13349-bib-0014] Glenton C , Colvin CJ , Carlsen B , Swartz A , Lewin S , Noyes J & Rashidian A (2013) Barriers and facilitators to the implementation of lay health worker programmes to improve access to maternal and child health: qualitative evidence synthesis. Cochrane Database Systematic Reviews, Issue 10, Art. No.: Cd010414. 10.1002/14651858.CD010414.pub2 PMC6396344 24101553

[jocn13349-bib-0015] Hannes K (2011) Chapter 4: Critical appraisal of qualitative research In: Noyes J, Booth A, Hannes K, Harden A, Harris J, Lewin S & Lockwood C (eds), Supplementary Guidance for Inclusion of Qualitative Research in Cochrane Systematic Reviews of Interventions. Version 1 (updated August 2011). Cochrane Collaboration Qualitative Methods Group. Available at: URL http://cqrmg.cochrane.org/supplemental-handbook-guidance (accessed 30 July 2015).

[jocn13349-bib-0016] Joshi R , Alim M , Kengne AP , Jan S , Maulik PK , Peiris D & Patel AA (2014) Task shifting for non‐communicable disease management in low and middle income countries – a systematic review. PLoS ONE 9, e103754. 25121789 10.1371/journal.pone.0103754 PMC4133198

[jocn13349-bib-0017] Kredo T , Adeniyi FB , Bateganya M & Pienaar ED (2014) Task shifting from doctors to non‐doctors for initiation and maintenance of antiretroviral therapy. Cochrane Database Systematic Reviews, Issue 7, Art. No.: Cd007331. 10.1002/14651858.CD007331.pub3 PMC11214583 24980859

[jocn13349-bib-0018] Lawn JE , Blencowe H , Oza S , You D , Lee ACC , Waiswa P , Lalli M , Bhutta Z , Barros AJD , Christian P , Mathers C & Cousens SN (2014) Progress, priorities, and potential beyond survival. The Lancet 384, 189–205. 10.1016/S0140-6736(14)60496-7 24853593

[jocn13349-bib-0019] Lehmann U , Van Damme W , Barten F & Sanders D (2009) Task shifting: the answer to the human resources crisis in Africa? Human Resources for Health 7, 49. 19545398 10.1186/1478-4491-7-49 PMC2705665

[jocn13349-bib-0020] Moxon SG , Lawn JE , Dickson KE , Simen‐Kapeu A , Gupta G , Deorari A , Singhal N , New K , Kenner C & Bhutani V (2015) Inpatient care of small and sick newborns: a multi‐country analysis of health system bottlenecks and potential solutions. BioMed Central Pregnancy and Childbirth 15, S7. 26391335 10.1186/1471-2393-15-S2-S7 PMC4577807

[jocn13349-bib-0021] NANN (2009) Position Statement #3009 – Minimum RN Staffing in NICUs. National Association Neonatal Nurses USA, Chicago.

[jocn13349-bib-0022] NCK (2014) Kenya Nursing Workforce Report – The Status of Nursing in Kenya, 2012. Nursing Council of Kenya, Nairobi.

[jocn13349-bib-0023] Noyes J & Lewin S (2011) Chapter 6: Supplemental Guidance on Selecting a Method of Qualitative Evidence Synthesis, and Integrating Qualitative Evidence with Cochrane Intervention Reviews In: Noyes J, Booth A, Hannes K, Harden A, Harris J, Lewin S & Lockwood C (eds), Supplementary Guidance for Inclusion of Qualitative Research in Cochrane Systematic Reviews of Interventions. Version 1 (updated August 2011). Cochrane Collaboration Qualitative Methods Group. Available at: URL http://cqrmg.cochrane.org/supplemental-handbook-guidance (accessed 30 July 2015).

[jocn13349-bib-0024] Pawson R , Greenhalgh T , Harvey G & Walshe K (2005) Realist review–a new method of systematic review designed for complex policy interventions. Journal of Health Services Research & Policy 10(Suppl. 1), 21–34. 16053581 10.1258/1355819054308530

[jocn13349-bib-0025] Thomas J & Harden A (2008) Methods for the thematic synthesis of qualitative research in systematic reviews. BioMed Central Medical Research Methodology 8, 45. 18616818 10.1186/1471-2288-8-45 PMC2478656

[jocn13349-bib-0026] Wakaba M , Mbindyo P , Ochieng J , Kiriinya R , Todd J , Waudo A , Noor A , Rakuom C , Rogers M & English M (2014) The public sector nursing workforce in Kenya: a county‐level analysis. Human Resources for Health 12, 6. 24467776 10.1186/1478-4491-12-6 PMC3913960

[jocn13349-bib-0027] WHO (2008) Task Shifting: Global Recommendations and Guidelines. Members Document. (WHO ed.). World Health Organization, Geneva.

[jocn13349-bib-0028] WHO (2012) WHO Recommendations: Optimizing Health Worker Roles to Improve Access to Key Maternal and Newborn Health Interventions through Task Shifting. World Health Organization, Geneva. 23844452

[jocn13349-bib-0029] WHPA (2008) Health Professions Demand Strong Principles for Task Shifting. World Health Professions Alliance, Paris.

[jocn13349-bib-0030] Yaya Bocoum F , Kouanda S , Kouyate B , Hounton S & Adam T (2013) Exploring the effects of task shifting for HIV through a systems thinking lens: the case of Burkina Faso. BioMed Central Public Health 13, 997. 24148691 10.1186/1471-2458-13-997 PMC4016414

[jocn13349-bib-0031] Baine SO & Kasangaki A (2014) A scoping study on task shifting; the case of Uganda. BioMed Central Health Services Research 14, 184. 24754917 10.1186/1472-6963-14-184 PMC4036592

[jocn13349-bib-0032] Callaghan‐Koru JA , Gilroy K , Hyder AA , George A , Nsona H , Mtimuni A , Zakeyo B , Mayani J , Cardemil CV & Bryce J (2012) ‘Health workers' and managers' perceptions of the integrated community case management program for childhood illness in Malawi: the importance of expanding access to child health services'. American Journal of Tropical Medicine and Hygiene, 87(Suppl. 5), 61–68. 23136279 10.4269/ajtmh.2012.11-0665 PMC3748524

[jocn13349-bib-0033] Cataldo F , Kielmann K , Kielmann T , Mburu G & Musheke M (2015) Deep down in their heart, they wish they could be given some incentives': a qualitative study on the changing roles and relations of care among home‐based caregivers in Zambia. BioMed Central Health Services Research 15, 1. 25627203 10.1186/s12913-015-0685-7 PMC4324023

[jocn13349-bib-0034] Cumbi A , Pereira C , Malalane R , Vaz F , McCord C , Bacci A & Bergstrom S (2007) Major surgery delegation to mid‐level health practitioners in Mozambique: health professionals' perceptions. Human Resources for Health 5, 27. 18062808 10.1186/1478-4491-5-27 PMC2235883

[jocn13349-bib-0035] Dambisya YM & Matinhure S (2012) Policy and programmatic implications of task shifting in Uganda: a case study. BioMed Central Health Services Research 12, 61. 22409869 10.1186/1472-6963-12-61 PMC3352120

[jocn13349-bib-0036] Ferrino P , Sidat M , Goma F & Dussault G (2012) Task‐shifting: experiences and opinions of health workers in Mozambique and Zambia. Human Resources for Health 10, 34. 22985229 10.1186/1478-4491-10-34 PMC3515799

[jocn13349-bib-0037] Ledikwe J , Kejepula M , Maupo K , Sebetso S , Thekiso M , Smith M , Mbayi B , Houghton N , Thankane K , O'Malley G & Semo B (2013) Evaluation of a Well‐Established Task‐Shifting Initiative: The Lay Counselor Cadre in Botswana. PLoS ONE 8, e61601 23585912 10.1371/journal.pone.0061601 PMC3621674

[jocn13349-bib-0038] Mpofu M , Semo B , Grignon J , Lebelonyane R , Ludick S , Matshediso E , Sento B & Ledikwe J (2014) Strengthening monitoring and evaluation (M&E) and building sustainable health information systems in resource limited countries: lessons learned from an M&E task‐shifting initiative in Botswana. BioMed Central Public Health 14, 1032. 25281354 10.1186/1471-2458-14-1032 PMC4192275

[jocn13349-bib-0039] Munga M , Kilima S , Mutalemwa PP , Kisoka W & Malecela M (2012) Experiences, opportunities and challenges of implementing task shifting in underserved remote settings: the case of Kongwa district, central Tanzania. BioMed Central International Health and Human Rights 12, 27 23122296 10.1186/1472-698X-12-27 PMC3503551

[jocn13349-bib-0040] Ochieng B , Akunja E , Edwards N , Mombo D , Marende L & Kaseje DC (2014) Perceptions of health stakeholders on task shifting and motivation of community health workers in different socio demographic contexts in Kenya (nomadic, peri‐urban and rural agrarian). BioMed Central Health Services Research 14, S4. 25079588 10.1186/1472-6963-14-S1-S4 PMC4108867

[jocn13349-bib-0041] Smith S , Deveridge A , Berman J , Negin J , Mwambene N , Chingaipe E , Putchalski Ritchie LM & Martiniuk A (2014) Task‐shifting and prioritization: a situational analysis examining the role and experiences of community health workers in Malawi. Human Resources for Health 12, 24. 24885454 10.1186/1478-4491-12-24 PMC4014628

[jocn13349-bib-0042] Spies LA (2014) An Exploratory Descriptive Study on Task Shifting. University of Texas at Arlington, Unpublished. Available online at: https://uta-ir.tdl.org/uta-ir/bitstream/handle/10106/24464/Spies_uta_2502D_12536.pdf?sequence=1

[jocn13349-bib-0043] Yaya Bocoum F , Kouanda S , Kouyate B , Hounton S & Adam T (2013) Exploring the effects of task shifting for HIV through a systems thinking lens: the case of Burkina Faso. BioMed Central Public Health, 13, 997. 24148691 10.1186/1471-2458-13-997 PMC4016414

